# Preparation and Coagulation Performance of Polyaluminum Lanthanum Silicate Coagulant

**DOI:** 10.3390/ijerph20042793

**Published:** 2023-02-04

**Authors:** Jie He, Qixuan Song, Jian He

**Affiliations:** 1 Department of Environmental Science and Engineering, Fudan University, Shanghai 200433, China; 2 School of Life Sciences, Nanjing University, No.163 Xianlin Road, Nanjing 210023, China

**Keywords:** coagulant, water treatment, phosphate adsorption, polymer bridging

## Abstract

In order to address the growing problem of water pollution caused by the excessive discharge of contaminants and provide a better aquatic ecosystem for the public, increasing attention has been paid to the harmlessness and efficiency of coagulation. In this study, polyaluminum lanthanum silicate (PALS) was synthesized through co-polymerization as a novel coagulant to treat wastewater. FTIR, XRD, and SEM were used to analyze the morphology and structure of the material, which further confirmed that the PALS was successfully synthesized. The results indicated that PALS had a great performance in the treatment of a kaolin–humic acid suspension under the optimal synthesis conditions with Al/Si = 3, La/Si = 0.1, and basicity = 0.7. Compared with conventional coagulants, PALS exhibited a better performance at a low coagulant dose and could achieve a good removal effect for an ultraviolet wavelength less than 254 nm (UV_254_) (83.87%), residual turbidity (0.49 NTU), and dissolved organic carbon (DOC) (69.57%) at the optimal conditions. Additionally, the PALS showed a better effect on phosphate removal than other coagulants did, where the removal efficiency could reach 99.60%. Charge neutralization and adsorption bridging were the potential wastewater treatment mechanisms employed by the PALS, which showed varied contributions under different pH levels. The results indicated that PALS can be a promising coagulant in water treatment.

## 1. Introduction

Due to the excessive discharge of contaminants and lack of strict management, more and more areas have been suffering from increasing water pollution problems. An increasing number of emerging contaminants have been found to pose a serious threat to human health. Owing to its low costs, high efficiency, and convenient operation, coagulation is one of the most widely used methods to remove contaminants effectively, including suspended colloids, organic matter, and heavy metals in both drinking water and wastewater treatments [1,2]. The application of coagulation covers various industries such as the pulp and paper processing, pharmaceutical, cosmetics, food, mineral processing, metalworking, and textile industries [3,4]. The performance of coagulants determines coagulation efficiency to a large extent in water treatment [5,6,7]. Traditional inorganic coagulants such as polyaluminum chloride (PAC) have been widely used over the years as they can effectively remove suspended particles and contaminants by coagulation and sedimentation. However, they have been a concern for several years because the high concentration of residual aluminum in the aquatic environment may damage the human nervous system and even cause Alzheimer’s disease, dialysis encephalopathy, and presenile dementia [8,9,10,11]. Fe-based coagulants such as ferric chloride and polyferric sulfate are also undesirable as they would inevitably lead to an increase in turbidity and affect the color of effluent. Organic coagulants, such as polyacrylamides and poly-dimethyl-diallyl-ammonium chloride, have the advantages of lower dosage and volume of sludge production. Unfortunately, their toxicity and high cost limit their wide application. Although various coagulants have been applied in water purification, there is still a pressing need for more efficient and eco-friendly coagulants.

In order to meet the increasing market demands and environmental needs, hybrid material coagulants have been gradually investigated as they can offer tremendous potential with the synergetic effects of various components [12]. The addition of effective components leads to the formation of functional chemical groups and strengthens aggregating ability [13]. Polysilicic acid has been used to synthesize hybrid material coagulants because of its unique properties. Polysilicic acid can form into various species, including dimer (Si_2_O_3_(OH)_4_^2−^), trimer (Si_3_O_5_(OH)_5_^3−^), tetramer (Si_4_O_8_(OH)_4_^4−^), and polymer at different pH values [14,15]. With the addition of polysilicic acid, the molecular weight of coagulants can be increased, significantly improving their bridging capacity and helping small flocs aggregate and form bigger and denser flocs. Previous studies have found that polysilicate metal composite coagulants have excellent polymer bridging and sweep abilities in water treatment [14,16,17]. Through electrostatic interaction and complexation, metal cations can connect with polysilicic acid and form a stable structure [18]. The coagulation efficiency is further improved by the synergetic effect of metal cations and silicate groups. Some studies have focused on combining polysilicic acid with aluminum and irons [19]. Polysilicic–metal coagulants are found to be more adaptive to pH values and temperature. It was reported that polyferric silicate sulfate (PFSiS) showed a great performance in dealing with color and oil, even at low temperatures. Meanwhile, the risk of residual metal is also reduced, which is friendly to the environment [20,21,22].

Most studies on coagulants focus more on the removal of turbidity and organic matter and have rarely taken phosphorous removal into consideration. Eutrophication and harmful algal blooms caused by excessive discharge of phosphorous are developing into a global concern which may pose serious threats to the aquatic ecology, ecosystem, and public health [23,24,25]. It is of great environmental concern and sustainable significance to remove phosphorus from the aquatic environment and mitigate eutrophication. Various studies have been conducted on the development of modified materials to deal with severe phosphorus eutrophication [26,27,28]. Aluminum compounds have been in use for the removal of phosphate. However, the desorption of phosphorous under alkaline conditions and ecological effects limit the use of aluminum compounds. Lanthanum has shown a marvelous performance in attenuating phosphorus through its strong affinity to PO_4_^3−^ [29,30]. Additionally, lanthanum-modified materials are highly selective for phosphorus and widely adaptable to pH values [31]. Furthermore, lanthanum ions are highly positively charged and have great reactivity because of the unsaturated structure of the outer layer. Like other metal ions, lanthanum ions could form polynuclear hydroxyl complex ions through hydrolytic complexation in solution. However, lanthanum’s performance as a coagulant component is barely known. We hypothesize that introducing lanthanum into coagulants may improve the phosphate adsorption capacity through ligand exchange. Thus, synthesizing a novel coagulant with aluminum, lanthanum, and polysilicic acid through the co-polymerization method may retain the advantages of each component and further improve the coagulation efficiency and phosphate adsorption capacity.

In this context, one novel coagulant, polyaluminum lanthanum silicate (PALS), was synthesized through co-polymerization with aluminum salts, lanthanum salts, and polysilicic acid. The influences of the mole ratio of Al/Si and La/Si and basicity were considered to determine the optimal synthesis conditions. Fourier transform infrared spectroscopy (FTIR), X-ray diffraction (XRD), and scanning electron microscopy (SEM) were used to analyze the structure and morphology of PALS. The jar test was conducted to evaluate the coagulation performance of PALS compared with other coagulants in synthetic water. The purpose of this study was to synthesize a highly efficient coagulant for wastewater treatment and phosphate removal and explore its removal mechanism.

## 2. Materials and Methods

### 2.1. Materials

Aluminum chloride (AlCl_3_), lanthanum chloride (LaCl_3_·7H_2_O), sodium silicate (Na_2_SiO_3_·9H_2_O), sulfuric acid (H_2_SO_4_), sodium hydroxide (NaOH), polyaluminum chloride (PAC), kaolin, humic acid, potassium phosphate monobasic (KH_2_PO_4_), and sodium bicarbonate (NaHCO_3_) were purchased from Aladdin Reagent Co. Ltd. in Shanghai, China. All chemicals were of at least analytical reagent grade and used as received without further purification.

### 2.2. Preparation of Coagulants

The pH of sodium silicate solution with a concentration of 2.0% Si (*w*/*w*) was adjusted to 3 by adding H_2_SO_4_. Then, the obtained solution was stirred vigorously for 2 h at room temperature and left to stand for a certain time to form polysilicic acid (PSi). A certain amount of AlCl_3_ and LaCl_3_·7H_2_O was added to the solution with continuous stirring. After stirring for 1 h, NaHCO_3_ was added to the solution at a constant speed. After stirring for another 3 h and aging for 12 h, the products were dried at 70 °C for 12 h in a vacuum dryer. Polyaluminum silicate chloride (PASiC) was also obtained without the addition of lanthanum. Finally, the dried composites were ground and screened, and the obtained coagulant was called PALS.

### 2.3. Characterization of PALS

The crystal structure of the coagulants was characterized using an X-ray diffraction spectrometer with Cu Kα radiation and a 2θ range from 5° to 90°. The functional groups of PALS were determined using an FTIR spectrometer. The FTIR spectra were obtained from 4000 cm^−1^ to 400 cm^−1^ at a resolution of 4 cm^−1^. The sample was prepared by mixing with KBr at a ratio of 1:100 (*w*/*w*) and pressing into a film. The zeta (ξ) potential values of PALS and PAC at pH 3–8 were obtained using a zeta potential analyzer (Zetasizer Nano ZS90, Malvern, UK). The surface morphologies and elemental mapping of coagulants were analyzed by SEM and EDS, respectively.

### 2.4. Coagulation Experiments

Simulated water was used in the coagulation experiments, which was synthesized with kaolin, humic acid, and KH_2_PO_4_. Kaolin was used to simulate turbidity, with 3 g kaolin added into 1 L deionized water. Humic acid played the role of organic matter with the following procedure: 1 g humic acid was dissolved into 1 L deionized water with the pre-introduction of 0.4 g NaOH and then kept stirring for 30 min. Finally, the obtained solution was filtrated through a 0.45 μm Millipore filter. KH_2_PO_4_ was used to simulate total phosphorus (TP), and 1 g L^−1^ phosphate stock solution was prepared by adding 4.38 g KH_2_PO_4_ into 1 L deionized water. All the stock solutions were stored at 4 °C, and the experimental solution was diluted with phosphorus stock solution before use. The parameters of the simulated water are shown in Table 1.

The coagulation experiments were performed in 1.0-L plexiglass beakers with six paddles stirrers (ZR4-6 China) (Figure S1). The whole procedure can be divided into three parts: stirring with high speed, flocculation with low speed, and sedimentation. First, 1000 mL of the prepared synthetic water was added to six glasses (1.2 L), and a certain amount of coagulants was added to each glass. Then, starting with the high stirring rate at 350 rpm (G value = 265 s^−1^) for 1 min, the colloids and suspensions were well mixed with PALS and destabilized. The stirring rate was set to 70 rpm for flocculation, which corresponded to the stirring intensity G value of 50 S^−1^, and the flocculation lasted 10 min. Finally, the water samples below 3.00 cm from the surface were collected for subsequent measurements after settling for 20 min. Part of the water sample collected was directly used to determine turbidity, while the rest was filtered through a 0.45 μm pore size filter, and all filtrates were stored at 4 °C before the measurement of DOC concentration, phosphate concentration, and UV_254_.

Jar test experiments were designed based on response surface methodology (RSM), which was used to analyze the interactions between synthetic conditions and obtain the optimal solution through the responses. This method used a continuous variable surface model to evaluate the influences of synthetic conditions on residual turbidity (Y_1_) and UV_254_ (Y_2_). The analysis method of Box–Behnken design (BBD) was used to analyze the preparation of PALS. The analysis had three levels, and each level possessed three factors and two responses. In each group of the experiment, the factors of pH (X_1_) and dosage of coagulants (X_2_) were fixed, with another three independent variables: the mole ratio of Al/Si, the mole ratio of La/Si, and basicity (the relative amount of OH^−^ compared to metal ions) conducted in rotation. As shown in Table 2, when analyzing the factor of Al/Si (varying from 1 to 3), the mole ratio of La/Si and the basicity were determined to be 0.1 and 1, respectively. When it came to the mole ratio of La/Si (varying from 0.05 to 1), the mole ratio of Al/Si and the basicity were fixed to be 3 and 1, respectively. As for basicity (varying from 0.1 to 1), the mole ratios of Al/Si and La/Si were set to 3 and 0.1, respectively. Furthermore, during the whole process, the value of pH ranged from 3 to 8, and the dosage of coagulants ranged from 4 mg L^−1^ to 12 mg L^−1^. A quadratic model was used to analyze these results, and the most suitable second-order polynomial equations would be obtained as follows:

$$Y=\beta_{0}+\overset{n}{\underset{i=1}{\sum }}\beta_{i}X_{i}+\overset{n}{\underset{i=1}{\sum }}\beta_{ii}X_{i}^{2}+\sum^{\;}\underset{i<j}{\sum }\beta_{ij}X_{i}X_{j}+\varepsilon$$

where *β*_0_ is the offset term; *β_i_*, *β_ii_*, and *β_ij_* are linear coefficients, quadratic coefficients, and interactive coefficients, respectively; *Y* represents the response, including residual turbidity and UV_254_ removal efficiency; *ε* is the error; and *n* is the number of factors. All coefficients and corresponding analyses on the equations were obtained with Design Expert 10.0. Analysis of variance (ANOVA) was performed for each response to evaluate whether the observed differences were statistically significant. Values of “Prob > F” less than 0.05 indicated that model terms were significant. Regression coefficients (*R*^2^) were used to check the adequacy of the model, and *R*^2^ values higher than 0.80 were considered valid.

### 2.5. Analytical Methods

The phosphate concentration in all filtrates was analyzed using the molybdenum blue colorimetric method on an HACH DR600 spectrophotometer at a wavelength of 700 nm. The concentration of DOC was measured with a TOC-L CPH CN200 analyzer (Shimadzu, Japan). Turbidity was measured with a 2100Q turbidimeter (HACH Company, America). UV_254_ was measured with a standard method using a UV-5200 spectrophotometer at a wavelength of 254 nm with a 1 cm quartz cell.

## 3. Results and Discussion

### 3.1. Optimization of Preparation Conditions

The performance and efficiency of the PALS coagulants were greatly influenced by several synthesis conditions, including the mole ratio of Al/Si, the mole ratio of La/Si, and the basicity of the PALS. The analysis method of Box–Behnken design was used to determine the optimal synthesis conditions of PALS. Throughout the experiment, pH and dosage were two fixed factors, whereas the three influencing factors mentioned above were adjusted. The results of the ANOVA on residual turbidity and UV_254_ removal data are shown in Tables S1 and S2. The *p*-values of the model were all less than 0.05, while the *p*-values of lack of fit were greater than 0.05, which suggested that the model has good suitability.

According to the quadratic model, the most suitable second-order polynomial equations for residual turbidity and UV_254_ in terms of coded factors were obtained as follows:

(1) $$Y_{\;\mathrm{Residual}\;\mathrm{turbidity}}=3.60\;–6.22X_{1}+2.72X_{2}–7.86X_{3}-2.78X_{1}X_{2}+6.30X_{1}X_{3}-4.94X_{2}X_{3}+1.97X_{1}^{2}+4.01X_{2}^{2}+3.55X_{3}^{2}$$

(2) $$Y_{\;\mathrm{uv}254\;\mathrm{removal}\;\%}=79.96+6.34X_{1}+2.23X_{2}+5.92X_{3}+3.63X_{1}X_{2}-2.27X_{1}X_{3}+2.56X_{2}X_{3}-2.18X_{1}^{2}-7.34X_{2}^{2}-1.90X_{3}^{2}$$

For mole ratio of La/Si:

(3) $$Y_{\;\mathrm{Residual}\;\mathrm{turbidity}}=2.43\;–1.02X_{1}-2.22X_{2}–0.50X_{3}+0.53X_{1}X_{2}+0.094X_{1}X_{3}+0.43X_{2}X_{3}+0.29X_{1}^{2}+0.87X_{2}^{2}-0.50X_{3}^{2}$$

(4) $$Y_{\;\mathrm{uv}254\;\mathrm{removal}\;\%}=82.47+2.76X_{1}+4.41X_{2}-0.11X_{3}+1.66X_{1}X_{2}-0.64X_{1}X_{3}-0.07X_{2}X_{3}-2.13X_{1}^{2}-6.30X_{2}^{2}-1.16X_{3}^{2}$$

For basicity:

(5) $$Y_{\;\mathrm{Residual}\;\mathrm{turbidity}}=4.10\;–2.75X_{1}-5.85X_{2}–1.43X_{3}-0.16X_{1}X_{2}+0.58X_{1}X_{3}+1.06X_{2}X_{3}+1.46X_{1}^{2}+4.69X_{2}^{2}+0.094X_{3}^{2}$$

(6) $$Y_{\;\mathrm{uv}254\;\mathrm{removal}\;\%}=76.53+5.89X_{1}+7.66X_{2}+3.23X_{3}+1.57X_{1}X_{2}-0.28X_{1}X_{3}-0.64X_{2}X_{3}-3.37X_{1}^{2}-7.70X_{2}^{2}-0.78X_{3}^{2}$$

**Figure 1:**
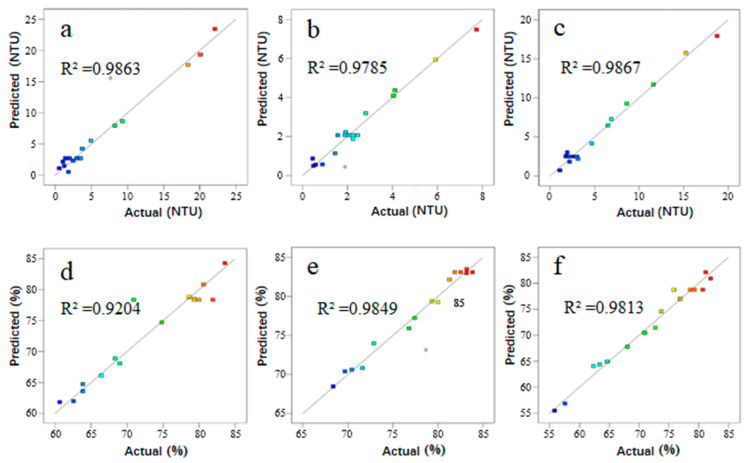
Plots of predicted values of the models versus actual values on different factors for residual turbidity: (**a**) Al/Si; (**b**) La/Si; (**c**) basicity and UV_254_ removal; (**d**) Al/Si; (**e**) La/Si; (**f**) basicity.

The coefficient of determination (*R*^2^) values of Equations (1)–(6) were all above 0.92, being equal to 0.9863, 0.9204, 0.9785, 0.9848, 0.9867, and 0.9813, respectively, thus demonstrating that the equations fitted pretty well to the data. Furthermore, the plots of the predicted values of the models versus the actual values for residual turbidity and UV_254_ removal are provided in Figure 1. Most of the points were distributed near the straight line, which indicated that making a relatively accurate prediction on turbidity and UV_254_ removal with the model was reliable.

**Figure 2:**
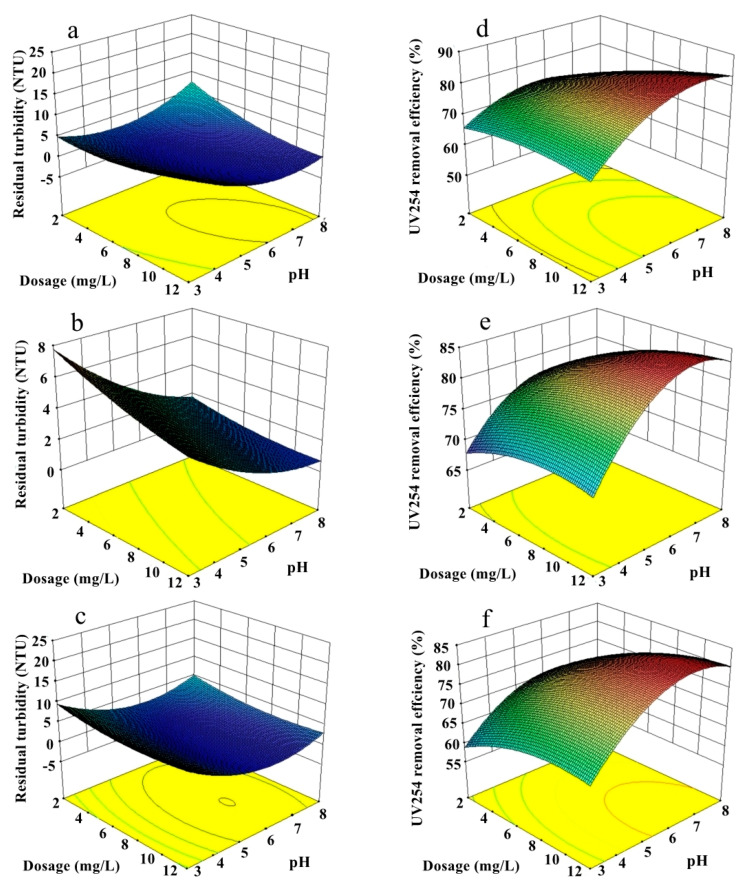
Effect of different factors on residual turbidity: (**a**) Al/Si; (**b**) La/Si; (**c**) basicity and UV_254_ removal; (**d**) Al/Si; (**e**) La/Si; (**f**) basicity.

The 3D response surfaces of residual turbidity and UV_254_ removal efficiency, which were obtained according to the quadratic BBD model, are displayed in Figure 2. According to the results, the turbidity and UV_254_ removal efficiency were strongly correlated with the content of Si. These results indicated that the addition of polysilicic acid could improve the bridging ability of the coagulant to a certain extent as it interacted with metal ions and formed complex hydroxy metal silicate, leading to the increase in molecular weight and size, which provided more absorption sites for absorbing the colloidal particles in solution [22]. However, various polymeric species (e.g., Si_2_O_3_(OH)_4_^2−^ and Si_3_O_5_(OH)_5_^3−^) are negatively charged which are consistent with the colloidal particles in the water and inhibit charge neutralization. Therefore, the negative effects brought by a high Si content cannot be ignored. In this study, the optimal mole ratio of Al/Si obtained from the model was three, which is much lower than that in previous studies [16,32,33]. This result suggested that the introduction of lanthanum in PALS would affect the polymerized product and improve the performance of PALS to some extent with the relatively low content of Al. With the increased content of lanthanum, the removal effect of turbidity and UV_254_ increased at low pH values of 3–5 and a dosage of 2–6 mg L^−1^, which indicated that the addition of lanthanum made the range of pH and dosage for PALS wider. However, considering its high cost and scarcity, the mole ratio of La/Si was determined to be 0.1.

The basicity (OH/(Al + La)) was another key point that determined the performance of PALS. The basicity was determined to be 0.7, and the lowest residual turbidity was 0.49 NTU. The UV_254_ removal efficiency could reach up to 83.87%. The addition of basic solution contributed to the formation of various polymeric products of Al—for example, Al(OH)_4_^−^, which was considered to be the precursor of Al_13_(Al_13_O_4_(OH)_24_^7+^)_,_ Al_2_(OH)_2_^4+^, and Al_3_(OH)_4_^5+^ [34], which possessed a larger molecular weight and size than monomeric Al. The charge neutralization ability and aggregation ability of the coagulant were greatly increased because of polymerization. Unfortunately, a continuous increase in basicity would be inevitably detrimental to the performance of PALS. These observations may be because the excess addition of OH^−^ made the formation of the polymer uncontrollable and decreased the content of efficient polymeric products such Al_13_, which led to the instability of the coagulant. Furthermore, the increase in pH was also responsible for the low performance under high basic solution, which may be because the metal ions were prone to precipitating into metal hydroxide rapidly in alkaline solution, resulting in a weakened coagulant performance.

### 3.2. Structure and Morphological Analysis

**Figure 3:**
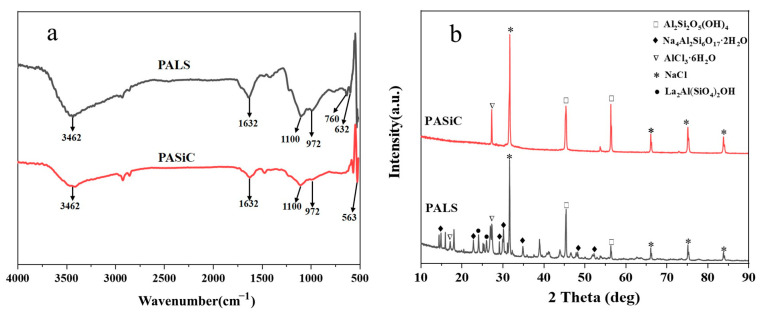
FTIR (**a**) and XRD patterns (**b**) for PALS and PASiC.

The FTIR spectra of PALS and PASiC are displayed in Figure 3a, whose mole ratios of Al:Si are 3:1 and 1:1, respectively. A strong and wide peak is observed at approximately 3462 cm^−1^ and a small peak is observed at approximately 1632 cm^−1^ in PALS and PASiC, which can be associated with the vibration of -OH. The peak at 3462 cm^−1^ is assigned to the stretching vibration of OH groups, whereas the other is owing to the bending vibration caused by the polymerization, absorption, and crystallization of water molecules in the coagulants [35,36]. Furthermore, the peak at 3462 cm^−1^ is significantly stronger in PALS than in PASiC, which may be attributed to the higher hydroxylation of Al and La increasing with the higher content of metal ions. Additionally, the peaks located at 1100 cm^−1^ correspond to the asymmetric stretching vibration of Si-O-Al and Si-O-La [37,38], which indicates that Si is partially replaced by Al and La in two coagulants. Furthermore, the peaks at 972 cm^−1^ and 563 cm^−1^ in PALS are attributed to Al-OH flexural vibration and Al-O stretching vibration, respectively [17,32,39]. The peaks between 632 cm^−1^ and 760 cm^−1^ are related to La-O and La-OH [38]. The FTIR results revealed that two novel polysilicate coagulants were successfully synthesized, which were covalently complex bound compounds by Al, La, and Si rather than simple physical mixing.

Figure 3b shows the XRD pattern of the two coagulants. Both coagulants exhibited diffraction peaks of NaCl, the by-product formed during the reaction between metal chloride and sodium salts. In terms of the size and structure of NaCl crystals, XRD is very sensitive to them, explaining the strong peaks in the pattern. AlCl_3_·6H_2_O was also found in the two coagulants, which was attributed to the crystallization of free aluminum ions. Both NaCl and AlCl_3_·6H_2_O have no effect on the performance of PALS. Furthermore, Al_2_Si_2_O_5_(OH)_4_, Na_4_Al_2_Si_6_O_17_·2H_2_O, and La_2_Al(SiO_4_)_2_OH diffraction peaks were observed in PALS, implying that it is not a simple mixture of materials but a complex chemical reaction. The results of XRD indicate that PALS is a unique inorganic polymer synthesized with polysilicon acid, aluminum chloride, and lanthanum chloride.

**Figure 4:**
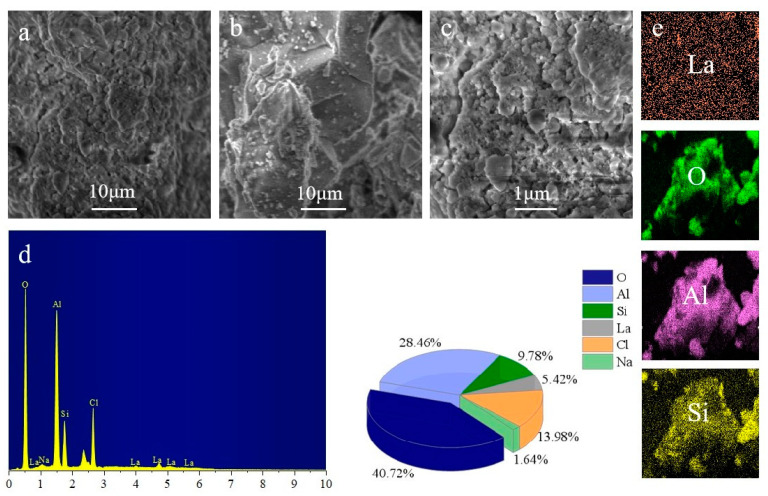
SEM photographs of PALS at low magnification (**a**), PASiC at low magnification (**b**), and PALS at high magnification (**c**); EDS spectrum of PALS (**d**); and mappings of PALS (**e**).

SEM was used to examine the optimized ratio of PALS to learn more about the morphology and structure of PALS. As depicted in Figure 4a, the PALS had an amorphous structure and a quite rough surface and was covered with a cluster structure on its surface. Compared with the relatively smooth surface of PASiC in Figure 4b, PALS possessed a higher surface area. The addition of lanthanum would influence the structure of coagulants through complexation. From the high magnifications in Figure 4c, PALS presented a dense and porous surface consisting of nanoscale particles whose radius was approximately 0.5 microns. The addition of aluminum and lanthanum ions combined with the long chain of silicate and continuous expansion in all directions ultimately formed this specific structure. This result makes PALS a promising coagulant and enhanced its adsorption performance in pollutants. Furthermore, EDS (Figure 4d) and MAP (Figure 4e) analyses were followed with SEM to determine the elements and specific atomic percentages of PALS. The existence of Al, Si, La, Na, Cl, and O suggests a good combination of raw materials, and the relative weight percentages of O, Al, Si, La, Cl, and Na were 40.72%, 28.46%, 9.78%, 5.42%, 13.98%, and 1.64%, respectively.

### 3.3. Effect of pH and Dosage

**Figure 5:**
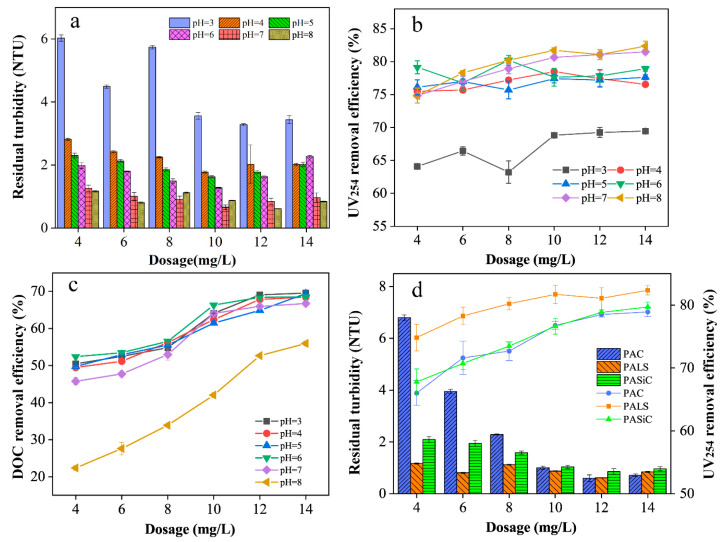
Effect of dosage and pH on residual turbidity (**a**), UV_254_ removal (**b**), and DOC removal (**c**) with PALS and comparison between PAC, PASiC, and PALS on residual turbidity and UV_254_ removal (**d**).

The coagulation performance of PALS was evaluated in simulated wastewater, whose parameters are shown in Table 1. It is clear that pH plays a vital role in affecting the efficiency of coagulation. As shown in Figure 5a, PALS showed a great performance in removing turbidity at pH 7–8 but a relatively poor performance at pH = 3 with residual turbidity of approximately 3.56 NTU to 6.03 NTU. The lowest residual turbidity was about 0.49 NTU with the dosage of 12 mg L^−1^ at pH = 8. With the continuous addition of coagulants, the residual turbidity reached the minimum and then increased a little at pH 3–7. It could be attributed to charge reversal in the solution, which led to the restabilization of colloids and suspensions.

As shown in Figure 5b,c, the highest removal efficiencies of UV_254_ and DOC for simulated water were 83.87% and 69.57%, respectively. With the increase in coagulant dosage, both UV_254_ and DOC removal efficiencies increased gradually at dosages of 4–10 mg L^−1^ but did not significantly improve over 10 mg L^−1^. When the pH varied from 3 to 8, UV_254_ removal demonstrated the same tendency as turbidity. The UV_254_ removal efficiency with different coagulant dosages at pH = 3 was only approximately 64.52–69.68%, dramatically increasing to 75.48–82.36% at pH = 4–8. The results indicated that the colloids and macromolecular organic matter may share a similar removal mechanism, while the difference was that the removal of macromolecular organic matter was not subject to excessive dosing. However, the DOC removal efficiency pattern at different pH values was completely opposite to that of UV_254_ removal. The DOC removal efficiency at pH 8 was only approximately 52% with the dosage of 14 mg L^−1^, whereas it improved significantly at pH 3–7, with a removal efficiency up to approximately 70%. The results suggested that alkaline conditions were not conducive to the removal of dissolved organic matter, while acidic conditions were not conducive to the removal of macromolecular organics and colloids. It may be because dissolved organic matter was more likely to be adsorbed through charge neutralization. When the pH was low, aluminum and lanthanum were in the form of Al^3+^ and La^3+^, and there was a lower polymerization degree coordination of Me^3+^ (metal ions) with hydroxyl in solution surrounded with large amount of H^+^, which was beneficial to the adsorption of dissolved organic matter.

As shown in Figure 5d, the coagulation performance of different coagulants was evaluated according to their abilities of turbidity and UV_254_ removal in simulated wastewater at pH = 7. PAC was purchased from Aladdin Reagent Co. Ltd. in Shanghai, of which Al_2_O_3_(wt%) = 30, B = 0.8, pH = 5. PASiC (Al/Si = 3, B = 0.8) was synthesized without the introduction of lanthanum. The stirring time and intensity were kept the same, as mentioned in Section 2.4. PAC performed worst at a low dosage with its turbidity over 3.95 NTU. With the continuous increase in coagulant dosage, PAC, PASiC, and PALS all showed a great performance in removing colloids, and the lowest turbidity was 0.59 NTU, 0.62 NTU, and 0.85 NTU, respectively. PALS exhibited a better performance in UV_254_ removal than the other coagulants at all dosages, and the best removal efficiency was 82.36%. It may be attributed to the addition of lanthanum having further improved the affinity of the coagulant to organic matter and facilitated the removal efficiency by forming coprecipitation with Al and polysilicic acid. The results above suggest that lanthanum has a promising effect in water treatment as a coagulant.

**Figure 6:**
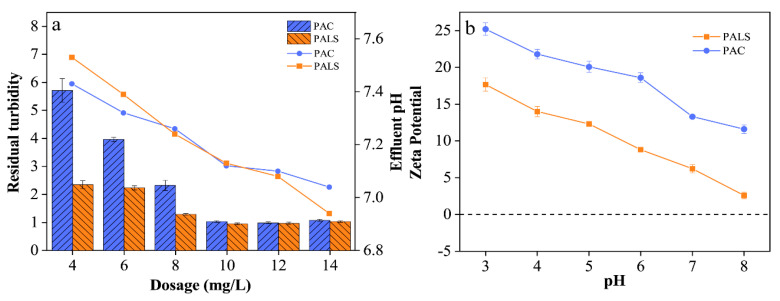
Residual turbidity and effluent pH of raw water from Ri Hu by PAC and PALS (**a**); zeta potential of PAC and PALS at pH 3–8 (**b**).

Figure 6a compares the coagulation performance of PALS and PAC in raw water from Ri Hu with different coagulant dosages. PALS performed better at low dosages, and the lowest turbidity was 0.88 NTU. PALS had more of an effect on effluent pH, which may be attributed to the introduction of polysilicic acid and sufficient hydrolysis of Al^3+^ and La^3+^. Although PAC was more positively charged than PALS, as shown in Figure 6b, favoring a stronger charge neutralization ability in coagulation, PALS performed better in that it may have a promising adsorption bridging and sweep trapping ability and work together to synergize water treatment.

### 3.4. Phosphate Removal

**Figure 7:**
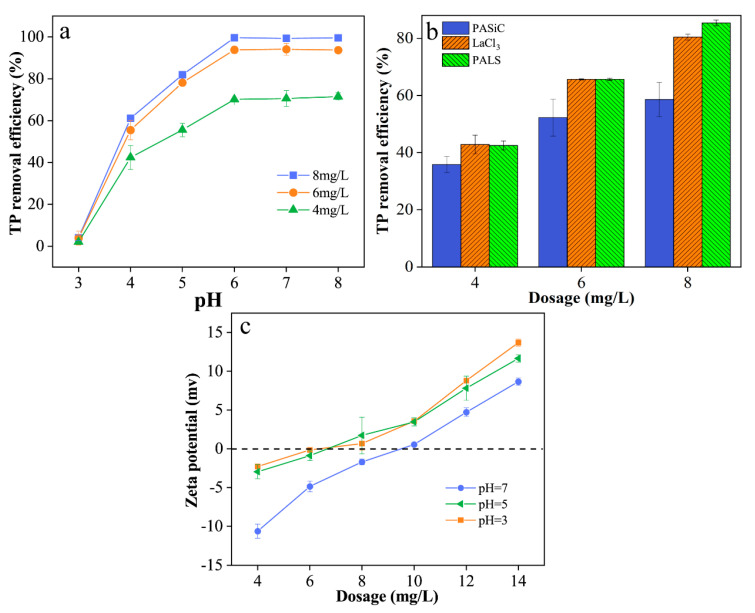
TP removal efficiency at pH = 3–8 by PALS with initial phosphate concentration = 2 mg L^−1^ (**a**); TP removal efficiency by PASiC, PALS, and LaCl_3_ at dosages of 4–8 mg L^−1^ with initial phosphate concentration = 5 mg L^−1^ and pH = 7.8 (**b**); zeta potential of flocs at dosages of 4–14 mg L^−1^ with PALS (**c**).

The removal of phosphate with PALS at different pH levels (3–8) was investigated with an initial phosphate concentration = 2 mg L^−1^, and the results are shown in Figure 7a. The removal of phosphate was greatly dependent on pH. The TP removal efficiency dramatically increased with the pH increasing from 3 to 6 and was maintained at a high level at pH 6–8. The uptake capacity continuously increased with the increase in the dosage of PALS from 4 mg L^−1^ to 8 mg L^−1^, and the maximum dephosphatization capacity at pH = 6 and a dosage of 8 mg L^−1^ could reach up to 99.6%. According to previous studies, the change in pH affects the form of lanthanum in solution and the chemical form of phosphate [40]. The main species of phosphate were H_2_PO_4_^−^ at pH 3–6 and HPO_4_^2−^ at pH 6–8. These results indicated that PALS may have a stronger affinity with H_2_PO_4_^−^ than HPO_4_^2−^, which is consistent with previous studies [41,42]. Notably, PALS had little effect on phosphate at pH = 3, possibly because lanthanum ions formed the complex of La(H_2_O)_n_^3+^ during the hydrolysis process at low pH values, in which water molecules surrounded the lanthanum ions as the center [43]. The water molecules were arranged in a certain geometric pattern, which packaged the lanthanum ions and prevented the lanthanum ions from complexing with phosphate.

Figure 7b compares the phosphate removal efficiency of PASiC, LaCl_3_, and PALS with an initial phosphate concentration of 5 mg L^−1^. Both LaCl_3_ and PALS exhibited a promising performance in dephosphorization, and the maximum removal efficiency was 80.41% and 85.34% at a dosage of 8 mg L^−1^, respectively. It could be concluded that La had a better dephosphatization capacity than Al. With the increase in coagulant dosage, PALS seemed to show a better phosphate removal capacity than LaCl_3_. It may be because the higher molecular weight and polymerization degree of PALS provided more adsorption sites for combining with phosphate and forming insoluble precipitation. Compared with incorporating La into carrier materials as an adsorbent, the coagulation process seems to be a better alternative in that coagulation maximized its dephosphatization capacity (Kajjumba, G.W. et al. 2022 [38]). The results illustrated that the introduction of lanthanum would greatly improve the phosphate removal capacity, and PALS was a superb coagulant in dephosphorization.

### 3.5. Coagulation and Dephosphorization Mechanisms

**Figure 8:**
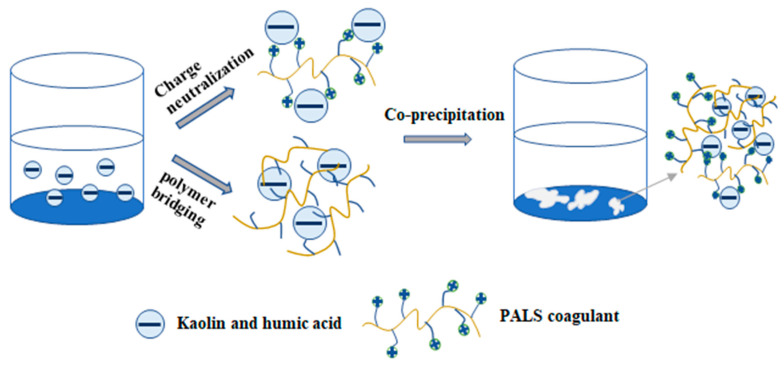
Schematic diagram of coagulation mechanisms.

Figure 8 summarizes the coagulation mechanisms and interactions in the system. When the pH was low, charge neutralization played a vital role in the coagulation process, as Al and La were present in acid solutions as Al^3+^ and La^3+^. However, in this study, charge neutralization seemed not to be an effective way to remove colloids, phosphate, and organic matter. Figure 7c shows the zeta potential of flocs at different dosages and pH levels and, it was interesting to find that the dosage at the + isoelectric point was not consistent with the dosage of optimal effects, which also explained that charge neutralization played a minor role in the system. When in a neutral or weakly alkaline solution, high polymerization of Al and La hydrolysis products was present in large amounts, which provided more adsorption sites and greatly improved adsorption bridging and sweep trapping. The improvement of the coagulation effect together with the increase in pH also indicated that adsorption bridging and sweep trapping dominated in the coagulation process. Additionally, it was also suggested that the dominant dephosphorization mechanism may be the direct adsorption of phosphates onto Al and La hydrolysis products, mainly referring to precipitation with insoluble Al(OH)_3_ and La(OH)_3_. Following the decrease in effluent pH, inner-sphere complexation may be another potential mechanism of phosphate removal through the formation of La-O-P and Al-O-P together with the release of H^+^.

## 4. Conclusions

This study developed a novel inorganic composite coagulant, polyaluminum lanthanum silicate, through co-polymerization with raw materials of aluminum chloride, lanthanum chloride, and sodium silicate. The optimal synthesis conditions were determined through response surface methodology, in which the mole ratio of Al/Si = 3, the mole ratio of La/Si = 0.1, and the basicity (OH/Al + La) = 0.7. PALS exhibited a relatively good performance at the optimal dosage and pH in the treatment of water, with residual turbidity below 1 NTU and a UV_254_ removal efficiency of over 80%. FTIR, XRD, and SEM were used to characterize and analyze the morphology and structure of PALS, confirming the formation of a novel composite polymer. Charge neutralization and adsorption bridging were considered to be the main mechanisms in the removal of colloids and organic matter, and the PALS had a stronger adsorption bridging effect owing to the introduction of polysilicic acid. The capture of phosphate was mostly dependent on electrostatic interaction and ligand exchange, and the residual phosphate concentration of 0.02 mg L^−1^ after treatment was far below the discharge standard of P. Compared with other coagulants, PALS showed great potential in phosphate adsorption owing to the modification of lanthanum. Above all, PALS is a promising coagulant in water treatment, especially with its brilliant performance in phosphate adsorption.

## Figures and Tables

**Table 1:** Parameters of simulated water.

Turbidity (NTU)	UV_254_ (cm^−1^)	DOC (mg L^−1^)	TP (mg L^−1^)	Temperature (°C)
28.6–30.2	0.145–0.152	29.68–30.42	2.02–2.06	24.5–25.2

**Table 2:** Coded levels for variables in BBD.

Factors	Level	
	−1	1
X_1_: pH	3	8
X_2_: Dosage	2	12
X_i_: Al/Si	1	3
X_i_: La/Si	0.05	1
X_i_: Basicity	0.1	1

## Supplementary Materials

The following supporting information can be downloaded at: https://www.mdpi.com/article/10.3390/ijerph20042793/s1, Figure S1: Schematic of the experimental rig; Table S1: ANOVA for PALS on residual turbidity data; Table S2: ANOVA for PALS on UV254 removal data.
