# Supplementary material for: Preparation and Coagulation Performance of Polyaluminum Lanthanum Silicate Coagulant
Source: Int J Environ Res Public Health. 2023 Feb 4;20(4):2793. doi: 10.3390/ijerph20042793 (PMC9957236; doi:10.3390/ijerph20042793)
Supplement: Supplementary file 1 [file ijerph-20-02793-s001.zip › ijerph-2145778-supplementary.pdf]

Preparation and Coagulation Performance of Polyaluminum Lanthanum Silicate Coagulant

Jie He [1], Qixuan Song [2,*] and Jian He [1,*]

[1] Department of Environmental Science and Engineering, Fudan University, Shanghai 200433, China

[2] School of Life Sciences, Nanjing University, No.163 Xianlin Road, Nanjing 210023, China

* Correspondence: sksqx@nju.edu.cn (Q.S.); hejian@fudan.edu.cn (J.H.); Tel./Fax: +86-21-31248911 (Q.S. & J.H.)

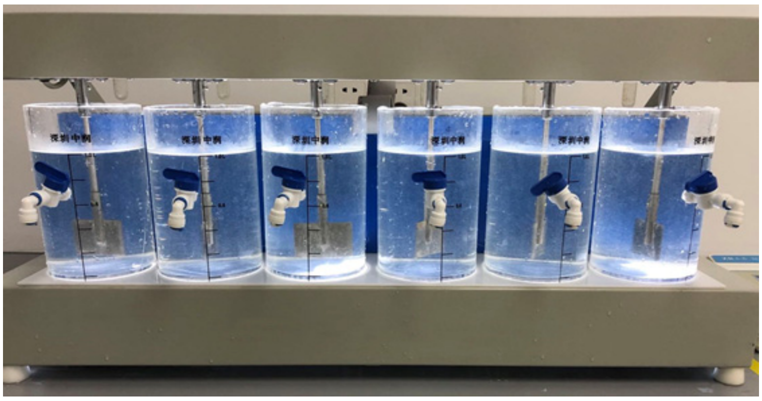

Figure S1. Schematic of the experimental rig.

Table S1. ANOVA for PALS on residual turbidity data.

Source	Al/Si			La/Si			Basicity		
	Mean Square	F Value	*p*-Value Prob > F	Mean Square	F Value	*p*-Value Prob > F	Mean Square	F Value	*p*-Value Prob > F
Model	87.34	56.15	< 0.0001	6.73	35.42	< 0.0001	53.67	50.66	< 0.0001
X_1	270.72	174.04	< 0.0001	8.14	42.87	0.0003	59.22	55.91	0.0001
X_2	52.68	33.87	0.0007	39.07	205.74	< 0.0001	153.93	145.32	< 0.0001
X_i	211.57	136.01	< 0.0001	2.21	11.62	0.0113	0.098	0.093	0.7697
$X_1 X_2$	31.44	20.21	0.0028	1.13	5.97	0.0446	11.29	10.66	0.0138
$X_1 X_i$	101.61	65.32	< 0.0001	0.040	0.21	0.6617	1.82	1.72	0.2312
$X_2 X_i$	63.82	41.03	0.0004	0.83	4.35	0.0756	0.025	0.023	0.8826
$X_1 X_1$	16.30	10.48	0.0143	0.36	1.89	0.2113	19.08	18.01	0.0038
$X_2 X_2$	61.16	39.32	0.0004	2.88	15.15	0.0060	151.02	142.57	< 0.0001
$X_i X_i$	21.74	13.98	0.0073	1.32	6.95	0.0336	7.01	6.62	0.0369
Residual	1.56			0.19			1.06		
Lack of Fit	2.47	2.85	0.1692	0.28	2.40	0.2086	1.53	2.16	0.2355
R-Squared		0.9863			0.9785			0.9867	
Adj R-Squared		0.9688			0.9509			0.9696	
Pred R-Squared		0.8456			0.7694			0.8243	

Table S2. ANOVA for PALS on UV_{254} removal data.

Source	Al/Si			La/Si			Basicity		
	**Mean Square**	**F Value**	***p*-Value Prob > F**	**Mean Square**	**F Value**	***p*-Value Prob > F**	**Mean Square**	**F Value**	***p*-Value Prob > F**
Model	99.04	8.99	0.0042	53.67	50.66	< 0.0001	126.35	40.87	< 0.0001
X_1	281.35	25.53	0.0015	59.22	55.91	0.0001	184.25	59.60	0.0001
X_2	35.41	3.21	0.1161	153.93	145.32	< 0.0001	311.83	100.86	< 0.0001
X_i	120.07	10.90	0.0131	0.098	0.093	0.7697	113.67	36.77	0.0005
X_1 X_2	53.76	4.88	0.0629	11.29	10.66	0.0138	10.22	3.31	0.1118
X_1 X_i	13.17	1.20	0.3105	1.82	1.72	0.2312	1.59	0.52	0.4961
X_2 X_i	17.17	1.56	0.2520	0.025	0.023	0.8826	8.44	2.73	0.1426
X_1 X_1	20.04	1.82	0.2195	19.08	18.01	0.0038	43.26	13.99	0.0073
X_2 X_2	204.63	18.57	0.0035	151.02	142.57	< 0.0001	225.46	72.93	< 0.0001
X_i X_i	6.20	0.56	0.4776	7.01	6.62	0.0369	63.57	20.56	0.0027
Residual	11.02			1.06			3.09		
Lack of Fit	1. 68	0.093	0.9598	1.53	2.16	0.2355	2.96	0.93	0.5037
R-Squared	0.9204			0.9849			0.9813		
Adj R-Squared	0.8180			0.9654			0.9573		
Pred R-Squared	0.7952			0.8396			0.8517		
